# Silk Fibroin-*g*-Polyaniline Platform for the Design of Biocompatible-Electroactive Substrate

**DOI:** 10.3390/polym14214653

**Published:** 2022-11-01

**Authors:** Elsa Veronica Flores-Vela, Alain Salvador Conejo-Dávila, Claudia Alejandra Hernández-Escobar, Rocio Berenice Dominguez, David Chávez-Flores, Lillian V. Tapia-Lopez, Claudia Piñon-Balderrama, Anayansi Estrada-Monje, María Antonia Luna-Velasco, Velia Carolina Osuna, Erasto Armando Zaragoza-Contreras

**Affiliations:** 1 Centro de Investigación en Materiales Avanzados, SC, Miguel de Cervantes No. 120, Complejo Industrial Chihuahua, Chihuahua 31136, Mexico; 2 Consejo Nacional de Ciencia y Tecnología CONACYT-Centro de Investigación en Materiales Avanzados, SC, CIMAV, Miguel de Cervantes 120, Complejo Industrial Chihuahua, Chihuahua 31136, Mexico; 3 Facultad de Ciencias Químicas, Universidad Autónoma de Chihuahua, Chihuahua 31125, Mexico; 4 Centro de Innovación Aplicada en Tecnologías Competitivas, A.C. Calle Omega No. 201, Industrial Delta, León 37545, Mexico

**Keywords:** silk fibroin, polyaniline, electroactive system, cytotoxicity

## Abstract

The structural modification of biopolymers is a current strategy to develop materials with biomedical applications. Silk fibroin is a natural fiber derived from a protein produced by the silkworm (*Bombyx mori*) with biocompatible characteristics and excellent mechanical properties. This research reports the structural modification of silk fibroin by incorporating polyaniline chain grafts through a one-pot process (esterification reaction/oxidative polymerization). The structural characterization was achieved by ^1^H-NMR and FT-IR. The morphology was studied by scanning electron microscopy and complemented with thermogravimetric analysis to understand the effect of the thermal stability at each step of the modification. Different fibroin silk (Fib): polyaniline (PAni) mass ratios were evaluated. From this evaluation, it was found that a Fib to PAni ratio of at least 1 to 0.5 is required to produce electroactive polyaniline, as observed by UV-vis and CV. Notably, all the fibroin-*g*-PAni systems present low cytotoxicity, making them promising systems for developing biocompatible electrochemical sensors.

## 1. Introduction

Conducting polymers have contributed significantly to biomedical applications [1] because their properties change depending on the pH and temperature, the presence of an oxidizing or reducing agent, and the acidic or basic environment [2]. Polyaniline (PAni), in particular, has shown essential roles in biomedical applications, such as scaffolds for cell reproduction [3]. However, limited biocompatibility and the absence of biodegradability are drawbacks that restrict its use in applications in the field of biomedical materials and biosensors [4]. Consequently, the design of electroconductive-electroactive polymeric materials, especially copolymer systems [5] and graft copolymers [6], is a strategy for solving such disadvantages. The intrinsic properties are different according to the architecture, e.g., random copolymers, their conductive characteristics, and the dedoping point could be modified, depending on the specific requirements [7]. On the other hand, the graft copolymers and block copolymers architectures do not change the main chain conjugation; however, they can increase colloidal stability, surface adherence, and in general, the processability of the systems [8].

On the other hand, the silk fibroin (Fib) is the base protein of silkworm (*Bombyx mori*) cocoons, consisting of the following amino acid sequence: glycine-alanine-glycine-alanine-glycine-serine (GAGAGS). Fib is a biocompatible material with exceptional properties [9]; for example, high strength, slow degradation, and water-processable [10]. Furthermore, grafting polymers from Fib has been a strategy for developing biocompatible materials. For example, Boonpavanitchakul et al. reported that the development of Fib-*g*-PLA copolymers with an increase in processability due to Fib reduces the crystallinity of PLA [11]. In addition, Nong et al. found a methodology for coloring silk through graft polymerization of acrylamides utilizing Lacasse as a catalyzer [12]. Lastly, Zhou et al. studied the Fib-*g*-polyacrylic acid, a graft copolymer, as a scaffold for biomimetic mineralization of Ca/P solutions [13]. Therefore, Fib is a material with potential and value in biomedical applications [14].

The synergy of Fib with conductive polymers allows the design of biocompatible and biodegradable systems with electroactive and electroconductive properties, which have promising potential in the regeneration of electrically-active tissues such as nerve fibers [15]. Scaffolds for tissue engineering with electrical properties and high mechanical properties with nerve regeneration potential have also been studied [16]. The development of biocompatible electrodes is another field of interest, in which is possible to monitor electrophysiological tests, e.g., electrocardiograms or electroencephalograms [17]. In addition, Fib-PAni copolymers with electroconductive properties were used as resistive sensors for ammonium and acetaldehyde with remarkable reversibility [18]. Similarly, Hong et al. reported an outstanding electrical conductivity (0.6 S/cm) for a Fib copolymer in the form of yarn [19].

Moreover, electrochromic-electroactive properties have also been studied in electronic textiles based on Fib-PAni fibers obtained by electrospinning, allowing the design of materials with environmentally friendly and biocompatible characteristics [20]. Likewise, Li et al. reported filaments of a Fib-PAni copolymer obtained through the wet-spinning technique, using an ionic liquid obtained from shell wastes and formic acid, as the solvent. It was found that the electrical conductivity of the filaments depends on the PAni content [21].

In this work, the synthesis of Fib-*g*-PAni copolymers from a one-pot methodology is reported, consisting of consecutive Fischer esterification and oxidative polymerization. Different characterization techniques were employed to analyze in detail the esterification reaction between Fib and aminobenzoic acid, and grafting to polyaniline chains by oxidative polymerization. This copolymer is proposed as a biocompatible-electroactive platform for designing electrochemical sensors that could be in contact with living tissue. This first manuscript describes the method of synthesis and characterization of the copolymer, its electrochemical behavior, and a first approach to cytotoxicity studies.

## 2. Materials and Methods

### 2.1. Materials

Silkworm cocoons, sodium carbonate (Merk, St. Louis, MO, USA, >98%), nitric acid (Fermont, Monterrey, Nuevo Leon, Mexico), dimethylsulfoxide (DMSO) (Merk, St. Louis, MO, USA, >99%), 3-aminobenzoic acid (Merk, St. Louis, MO, USA, >98%), aniline (Merk, St. Louis, MO, USA, >99.5%), sulfuric acid (Merk, St. Louis, MO, USA, >98%), ammonium persulfate (APS) (Merk, St. Louis, MO, USA, 98%), triple distilled water.

### 2.2. Silk Degumming

When working with Fib, it is essential first to remove sericin because it covers the fibroin structure, precluding surface interactions with other compounds. The methodology commenced by cutting the cocoons into fragments of around 1 cm^2^. The degumming process was carried out in a reflux system with magnetic stirring. The cut cocoons (1 g) and 50 mL of sodium carbonate aqueous solution (20 mM) were placed inside the reflux system. The mixture was heated to boiling point under continuous stirring for 2 h. Subsequently, the liquid was withdrawn and replaced with another 50 mL of the sodium carbonate solution, followed by another 2 h under the same conditions. Finally, the product was filtered and washed with tridistilled water. The Fib obtained was dried at room temperature [22].

### 2.3. Synthesis of the Fibroin-g-Polyaniline Copolymer

**Figure 1:**
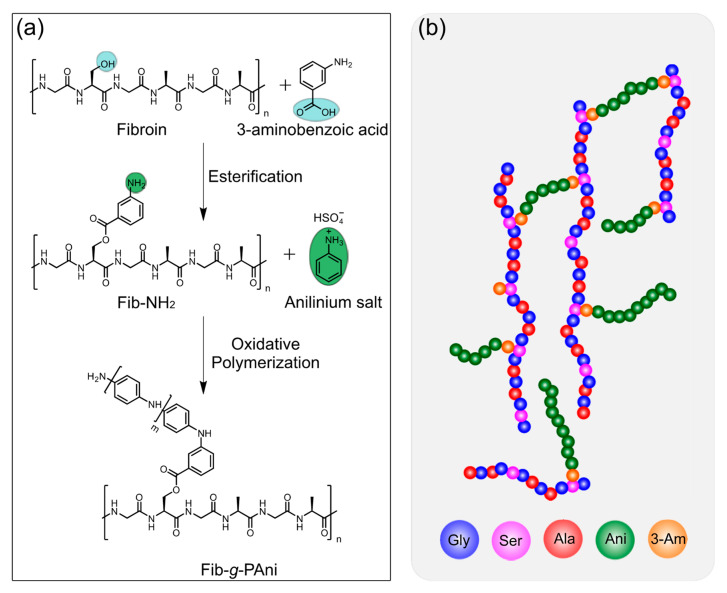
(**a**) Consecutive reactions for copolymer synthesis fibroin-*g*-polyaniline (Fib-*g*-PAni), and (**b**) proposed model of Fib-*g*-PAni.

The fibroin-*g*-polyaniline (Fib-*g*-PAni) copolymer was prepared through a one-pot methodology. Fischer esterification and oxidative polymerization were carried out consecutively. Figure 1 illustrates the reaction scheme and the proposed model of Fib-*g*-PAni.

#### 2.3.1. Fischer Esterification

The organic reaction was performed in a reactor provided with temperature control and magnetic stirring. DMSO (10 mL), Fib (1 g), and nitric acid (1 mL) were then added and stirred for 1 h at laboratory temperature. Next, 3-aminobenzoic acid (150 mg, 1.1 mmol) was added dropwise, and then the reactor was heated to 70 °C, maintaining magnetic stirring for 6 h. The product was called Fib-NH_2_.

It is worth saying that to carry out the characterization, Fib-NH_2_ was purified by dialysis in water (2 days) to remove the DMSO. Afterward, the compound was purified through a chromatographic column using an ethanol: ethyl ether (1:9) mixture as the mobile phase. After purification, the Fib-NH_2_ was an orange solid, and the reaction presented a yield of 35%.

#### 2.3.2. Oxidative Polymerization

The Fib-NH_2_ solution (without purification) was cooled to room temperature, and the anilinium salt (polyaniline precursor monomer) was added. Table 1 reports the experimental systems. APS was added in a molar ratio of 5:4, regarding the anilinium sulfate monomer. Both solutions were stirred separately and subsequently mixed. The polymerization was carried out at 60 °C for 6 h. After polymerization, the product was purified by three precipitation–centrifugation cycles. The precipitation was performed with 100 mL of sulfuric acid aqueous solution (H_2_SO_4_, 3 wt%) to prevent the dedoping effect. Finally, the product was washed with the same acid solution and dried at room temperature. The Fib-*g*-PAni was solid with a dark green color.

### 2.4. PAni Polymerization

The neat PAni homopolymer was synthesized in a flask equipped with a stirring bar. Firstly, 100 mL of H_2_SO_4_ (1 M) and aniline (10.737 mmol) were added. Once the anilinium salt was formed, the oxidizing initiator APS (13.42 mmol) was added. The solution was cooled at 4 °C for 24 h. The homopolymer, a dark green suspension, was purified by centrifugation at 5000 rpm. The solid was redispersed with H_2_SO_4_ (0.1 M) to prevent the dedoping process for three cycles. The yield of polymerization was 75%.

### 2.5. Cytotoxicity Assays on Fib-g-PAni

The cytotoxicity of the Fib-*g*-PAni 1:1, Fib-*g*-PAni 1:0.5, Fib-NH_2,_ and PAni was evaluated in NIH/3T3 fibroblast cells (ATCC^®^ CRL-1658™) through the MTT (methyl thiazole tetrazolium) assay. Just prior to the assay, the powdered copolymer and instruments were sterilized under UV light for 25 min (5 cycles of 5 min). Tested compounds were then dispersed in a culture media to reach 0.1, 0.5, 2, and 4 mg/mL of the Fib-*g*-PAni copolymers. For Fib-NH_2,_ the contents were 0.05, 0.33, 1, and 2 mg/mL and for PAni 0.03, 0.25, 1, and 2 mg/mL, respectively. For the assay, cells were cultured in a DMEM-F12 HAM media supplemented with 5% fetal bovine serum and incubated at 37 °C with 90% humidified/5% CO_2_/95% air. The media was replaced every 24–48 h until 80–90% confluence was reached for seeding treatments. NIH/3T3 cells were seeded in 96-well microplates at a density of 2 × 10^4^ cells/well (100 µL supplemented media) and incubated under identical conditions for 24 h. Old media was then replaced with 100 µL of treatment dilutions. A dilution of 0.4% SDS was parallel assayed as a positive control and cells not treated were used as reference controls. All treatments were carried out in triplicate, and the microplate was incubated for 24 h. After incubation, all media was removed, and cells were gently washed with PBS at pH 7.4. A total of 100 µL MTT of culture media (0.5 mg/mL) was then added to each well. The microplate was incubated for another 4 h. The media/MTT was removed, and 100 µL of acidified isopropanol was added to each well to dissolve the MTT metabolized by the cells. Finally, the plate was read at an absorbance of 570 nm using a microplate reader (Varioskan Lux VLBLATD2, Thermo Scientific, Waltham, MA, USA). Cell viability relative to untreated cells (reference control) was determined as follows.

Cell viability (%) = [(O.D. Test)/(O.D. Reference control)] × 100(1)

where O.D. (Test) and O.D. (Reference) represent the mean absorbance of the treated cells and the absorbance of the reference cells, respectively.

### 2.6. Characterization

The structural characterization of the products was performed by ^1^H-NMR spectroscopy, using the spectrophotometer (NMR Bruker Ascend 400 MHz, Billerica, MA, USA) at 400 MHz, and DMSO-d_6_ as the solvent. To solubilize Fib in DMSO-d_6_, adding 10 µL of nitric acid and sonication was necessary. The structural characterization was complemented with infrared spectroscopy (FT-IR) using a Fourier transform spectrophotometer (GX-FTIR, Perkin Elmer, Waltham, MA, USA). Spectra were obtained using the attenuated total reflectance (ATR) technique. The thermal stability of the copolymers was studied by thermogravimetric analysis (SDR Q600, TA Instruments, New Castle, DE, USA) with a sensitivity of 0.1 µg. The samples were heated from room temperature to 700 °C with a heating ramp of 10 °C min^−1^ in an air atmosphere.

Additionally, the absorption of all compounds in the UV and visible range was studied using a UV-VIS-NIR spectrometer (Evolution 220, Thermo Scientific, Waltham, MA, USA). In addition, a dispersion of the samples (1 mg) in 5 mL of distilled water and then sonicated 10 min, was utilized for analysis. The morphology of the Fib-*g*-PAni was observed using a field emission scanning electron microscope (JSM-7401F, Jeol Ltd., Akishima, Tokyo, Japan), operating in emission mode (FE-SEM).

The electroactivity properties were studied through a three-electrode system, employing as a reference electrode a Ag/AgCl electrode, a platinum plate (1 cm^2^) as the counter electrode, a glassy carbon electrode acting as a working electrode, and a potentiostat (Emstat3+ blue, PalmSense BV, Houten, The Netherlands). The electrochemical properties were explored by cyclic voltammetry with a potential window from −0.5 to 1.0 V, and the experiments were realized with H_2_SO_4_ (1 M) as an electrolyte.

## 3. Results and Discussion

The structural modification of Fib was developed through a Fisher esterification reaction between the 3-aminobenzoic acid and the serine present in Fib. The typical molar content of serine is 12.2% in fibroin [23]. Subsequently, the PAni chains were grafted, employing oxidative polymerization varying the ratio of the anilinium salt monomer and Fib-NH_2_, synthesizing four Fib-*g*-PAni copolymers.

### 3.1. Chemical Characterization

**Figure 2:**
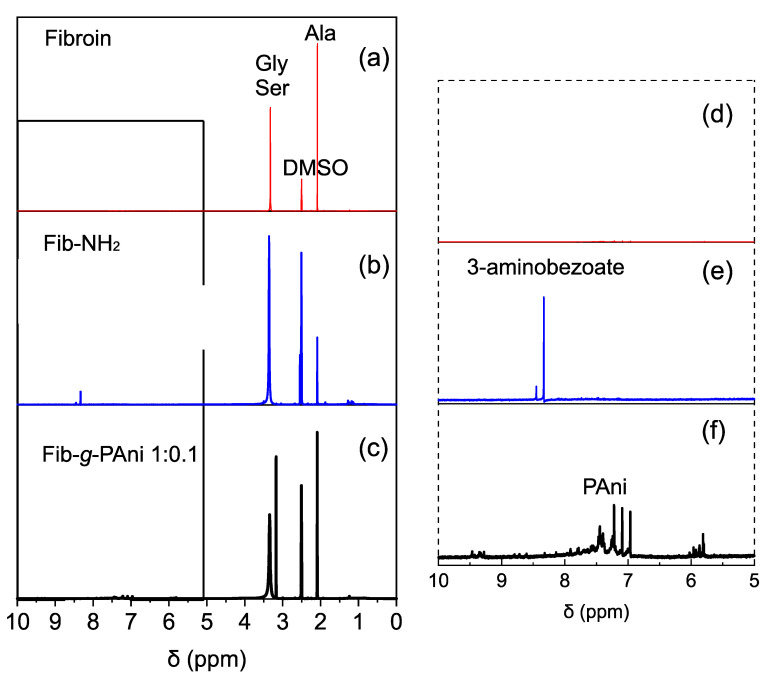
Proton NMR spectra (**a**–**c**) and zoom (**d**–**f**) from 5 to 10 ppm of these spectra. (**a**,**d**) Fibroin (Fib); (**b**,**e**) Fibroin esterified with aminobenzoate (Fib-NH_2_); (**c**,**f**) Fibroin-*g*-polyaniline (Fib-*g-*PAni).

The structural characterization of Fib-*g*-PAni and its predecessor compounds was performed employing ^1^H-NMR and FT-IR spectroscopy. Figure 2 displays the ^1^H-NMR spectra of Fib, Fib-NH_2,_ and Fib-*g*-PAni 1:0.1 (this copolymer was selected because the analogs with higher PAni contents are not soluble). The three spectra present the characteristic signals of glycine and serine at 3.4 ppm, and also the signal at 2.10 ppm corresponding to alanine of the fibroin structure [24]. The Fib-NH_2_ exhibits a new band at 8.3 ppm, corresponding to aromatic protons of the 3-aminobenzoate substituent product of Fisher esterification. Finally, the spectrum of Fib-*g*-PAni 1:0.1 displays typical signals between 7 and 8 ppm, corresponding to oligomers or PAni chains, and a new band appears around 3.1 ppm assigned to serine segments grafted with PAni chains.

**Figure 3:**
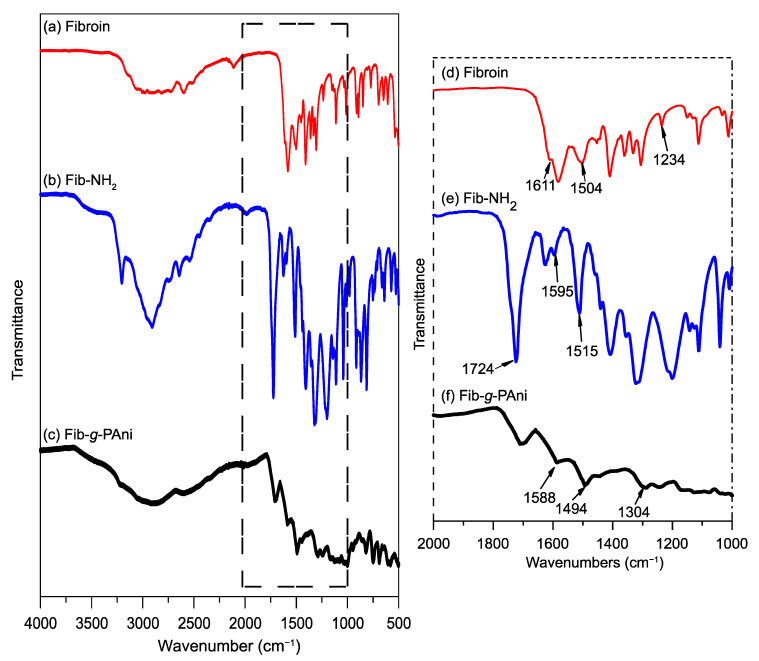
FT-IR spectra (**a**–**c**) and zoom (**d**–**f**) from 1000 to 2000 cm^−1^ of these spectra. (**a**,**d**) Fib, (**b**,**e**) Fib-NH_2_, and (**c**,**f**) Fib-*g*-Pani 1:0.5.

The FT-IR spectroscopy complemented the results of ^1^H-NMR. The degummed Fib presents the bands reported in the literature [18], see Figure 3. The spectrum shows signals at 1611 and 1504 cm^−1^ corresponding to the stretching vibration of the carbonyl group (C=O) and the bending vibration of the N–H bond of a secondary amine. Both signals are characteristic of the amide functional group in the Fib. In addition, the stretching vibration of the C–O bond of the alcohol group of serine and tyrosine is observed at 1234 cm^−1^ [25]. Compared with the Fib, the spectrum of Fib-NH_2_ has two new peaks. Specifically, the stretching vibration corresponding to the ester group (C=O) at 1724 cm^−1^, and the peak at 1595 cm^−1^ is assigned to the C=C bond of the aromatic ring of benzoate. The amine (N–H) vibration shifts to 1515 cm^−1^. Therefore, Fischer esterification between Fib and 3-amino benzoic acid was confirmed.

The spectrum of Fib-*g*-PAni 1:0.5 shows the characteristic signals of the PAni. For example, the vibrations corresponding to the quinoid and benzenoid rings were observed at 1588 and 1494 cm^−1^, respectively. A typical vibration of oxidative polymerization corresponding to C–N appears at 1304 cm^−1^. As noted, the peak height of the benzenoid structure is greater than the band of the quinoid structure, so it can be deduced that the PAni is in the emeraldine oxidation state [26].

### 3.2. Optical Activity

**Figure 4:**
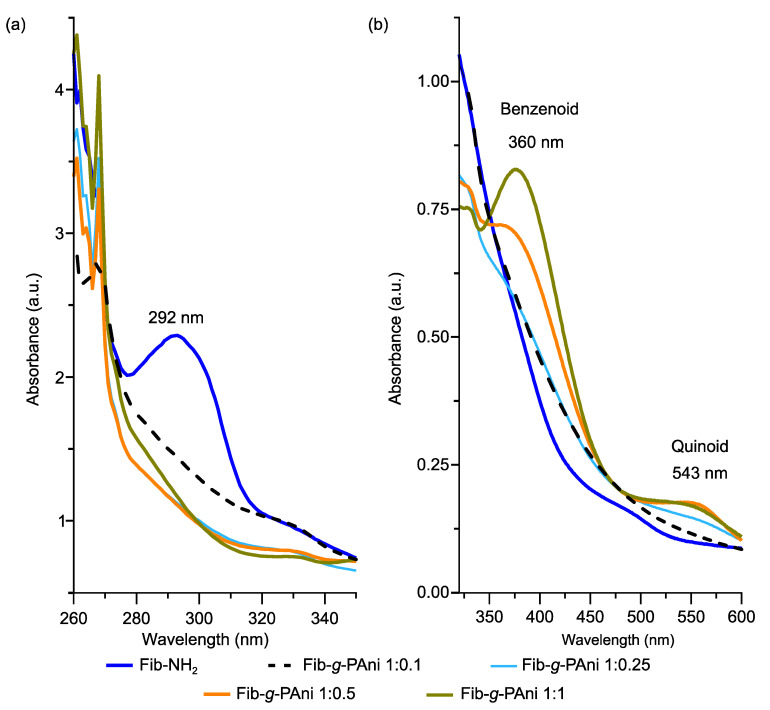
UV-Vis spectra of Fib-NH_2_ and Fib-*g*-PAni copolymers (**a**) UV-Vis spectra from 260 to 350 nm and (**b**) UV-Vis spectra from 340 to 600 nm.

Absorption studies in the UV-Visible range were conducted to understand band changes due to Fisher esterification and the formation of benzenoid and quinoid bands (oxidative polymerization of PAni). The esterification reaction between aminobenzoic acid and Fib produces a band at 292 nm, assigned to the π–π* transition of the aromatic ring of the aminobenzoate substituent. The esterification product was employed as grafting points for oxidative polymerization [27]. Nevertheless, the 292 nm band decreased after the polymerization process, due to the propagation of PAni chains and the change in the conjugation structure, see Figure 4a.

The Fib-*g*-PAni copolymers spectra with different mass ratios present different absorbances due to the formation of PAni chains, see Figure 4b. However, the Fib-*g*-PAni 1:0.5 and 1:1 spectra display the quinoid and benzenoid bands at 360 and 543 nm, respectively. The intensity and wavelength of absorption indicate the oxidation state of polyaniline (emeraldine base form) [28]. Therefore, a 2:1 ratio of Fib to aniline salt at least is required to produce PAni chains with a long enough degree of polymerization to produce an optical signal. At lower mass ratios only oligomers are produced [29]; nevertheless, they do not have the characteristic optical and electronic properties of PAni.

### 3.3. Thermal Stability

**Figure 5:**
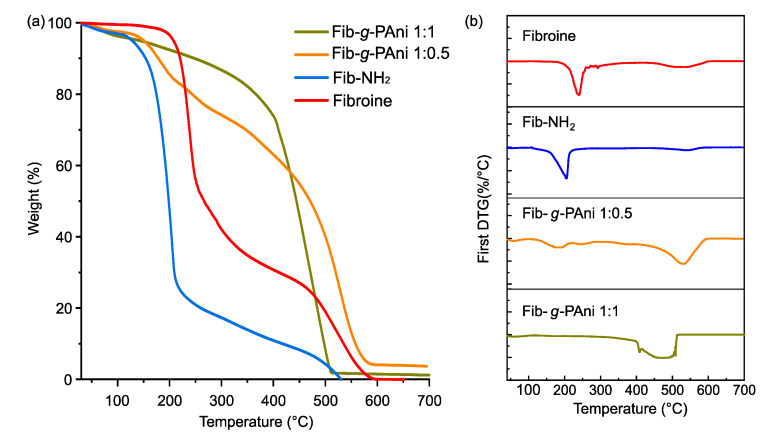
(**a**) Thermograms and (**b**) DTG of Fib, Fib-NH_2_, Fib-*g*-PAni 1:0.5 and Fib-*g*-PAni 1:1.

The thermal stability of the materials was determined by thermogravimetric analysis. Figure 5 displays the thermograms and first derivative (DTGA) of Fib, Fib-NH_2,_ and Fib-*g*-PAni 1:1 and Fib-*g*-PAni 1:0.5 copolymers. The Fib presents two stages of weight loss; the first is at 238 °C, and represents 70% of the sample weight. This stage is allocated to fragmented Fib chains produced by the degumming process [30]. The second step occurs at 530 °C, attributed to the Fib chain with a high molecular weight [31].

The thermal degradation of Fib-NH_2_ occurs at two stages, similar to Fib. The first occurs at 202 °C and is assigned to fragments of Fib hydrolyzed during Fisher esterification, due to the acid-catalyst-produced hydrolysis (chain degradation). This stage represents 80% of the total weight loss. Furthermore, the second occurs at 638 °C, with 20% of weight loss, corresponding to the Fib which is less hydrolyzed, and esterified with the aniline analog.

Fib-*g*-PAni 1:0.5 traces present three stages. The first weight loss was observed at 179 °C, assigned to organic low molecular weight compounds such as solvent or byproduct derivatives of one-pot synthesis. The second occurs at 259 °C, with a 20% of sample weight loss, corresponding to Fib chains with low PAni grafting [32]. The third stage occurs at 532 °C, with 80% of weight loss. In contrast to its intermediates, Fib-*g*-PAni 1:0.5 presents the main weight loss at around 500 °C. Likewise, Fib-*g*-PAni 1:1 only presents one weight loss stage, at around 470 °C. These findings suggest the thermal degradation of PAni [33,34,35].

### 3.4. Morphology

**Figure 6:**
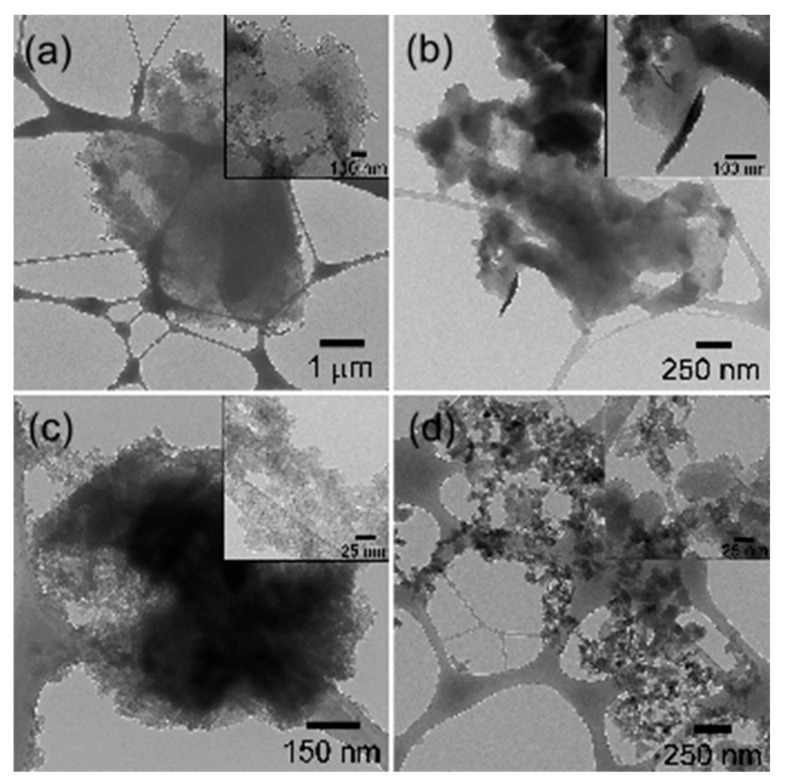
Micrographs of (**a**) Fib, (**b**) Fib-NH_2_, (**c**) Fib-*g*-PAni 1:0.5 and (**d**) Fib-*g*-PAni 1:1.

The morphological changes suffered by the Fib after each consecutive reaction were studied. Figure 6 illustrates the micrographs of degummed Fib, Fib-NH_2_, Fib-*g*-PAni 1:0.5, and Fib-*g*-PAni 1:1. The edge of the fibroin exhibits the fibrous nature of the material, see Figure 6a. The magnification of the image (inner box) illustrates this structure more evidently [36]. A change in morphology is evident when comparing Fib and Fib-NH_2_, see Figure 6b. In addition, the fibrous nature of the Fib disappears, suggesting that the incorporation of 3-aminobenzoic acid significantly alters the molecular arrangement [14].

Figure 6c,d correspond to the Fib-*g*-PAni samples with a mass ratio of 1:0.5 and 1:1, respectively. As noted, the PAni graft again produced important modifications on the Fib. The copolymers present particulate and globular structures for Fib-*g*-PAni 1:1 and Fib-*g*-PAni 1:0.5, respectively. The Fib-*g*-PAni 1:0.5 copolymer still retains a certain cohesion of the material; however, the aforementioned globular structure is perfectly observed under magnification. On the contrary, the Fib-*g*-PAni 1:1 copolymer has developed, to a greater extent, a particulate structure, and it is observed even in the magnification that the globular structures have, in turn, an internal structure.

### 3.5. Electroactivity

**Figure 7:**
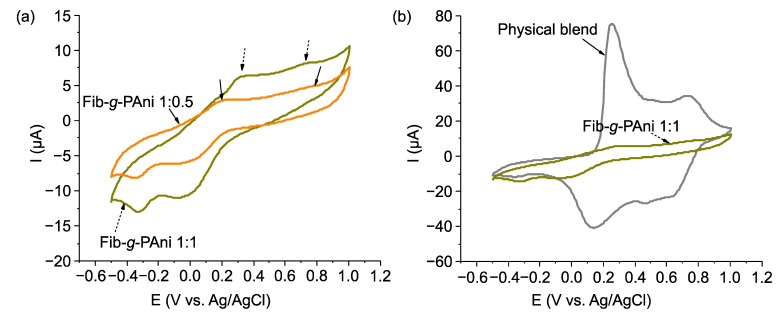
Voltamogramms of (**a**) Fib-*g*-PAni copolymers and (**b**) comparison between physical blends and graft copolymers.

To corroborate the electrochemical activity of the graft copolymers, cyclic voltammetry tests were carried out. Figure 7a shows the voltammograms of the Fib-*g*-PAni copolymers run in 0.5 M sulfuric acid electrolyte. Both graphs display the oxidation and reduction signals that the polyanilines regularly exhibit [37]. For example, for Fib-*g*-PAni 1:1, the first peak corresponds to the leucoemeraldine-emeraldine transition at 0.31 V (vs. Ag/AgCl). With the mass ratio of 1:0.5, the signal appears at 0.2 V (vs. Ag/AgCl). This difference could be due to the interaction between PAni and Fib or the degree of polymerization of PAni [38]. The second signal, corresponding to the emeraldine–pernigraniline transition, appears at 0.73 V (vs. Ag/AgCl) in both cases. The location of the signals is evidence of the electroactivity of the graft copolymers, which corroborates the results of the UV-vis study.

It is important to note that there are differences in electroactive properties between a physical mixture and a graft copolymer architecture. Figure 7b compares a physical blend of PAni-Fib, and the copolymer graft (Fib-*g*-PAni 1:1). It is noted that the intensity of peaks in the physical blend is significantly higher than the grafted copolymer. Thus, its general behavior is similar to conventional PAni, which indicates that interaction between Fib and PAni is only physical, and that the PAni in the blend has a higher molecular weight.

It is worth noting that in the graft PAni copolymers, the conjugation present in the conductive chain is not interrupted, which does not modify the electrochemical properties of PAni, which is different to other copolymer systems [8]. Furthermore, the electrochemical activity of PAni will depend on the polymerization process, dopant agent, and interaction with other polymers [38,39,40].

### 3.6. Cytotoxicity

**Figure 8:**
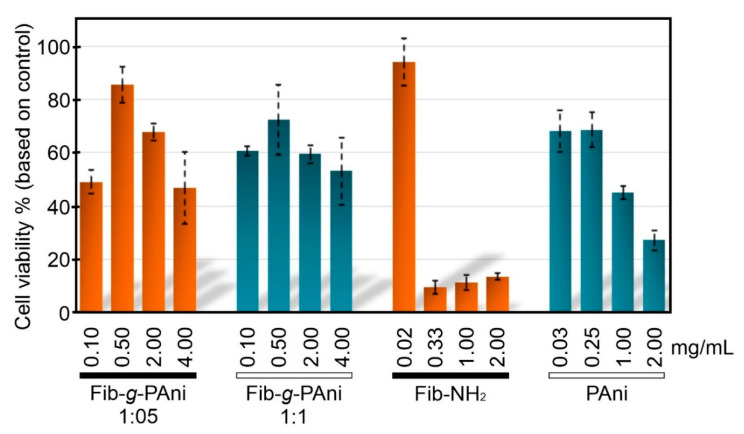
Cell viability of NIH/3T3 fibroblast cells treated with Fib-*g*-PAni (1:1 and 1:0.5), Fib-NH_2_, and PAni, taking the viability of untreated cells as a reference control.

It is well known that Fib displays biocompatibility properties, slow biodegradability, and low cytotoxicity [41]. Nevertheless, these properties are not extrapolated to structural modification and composites derived from Fib. For this reason, the cytotoxicity of Fib-*g*-PAni (1:1 and 1:05), Fib-NH_2_, and PAni were evaluated with NIH/3T3 fibroblast cells, measured at 24 h using the MTT assay [42], see Figure 8. The time for cell-tested viability was selected as the first approach. The use of fibroblasts is because they are the main active cells of connecting tissue, and are commonly used as models for viability/toxicity studies. In addition, the fibroblast presents an improved proliferation in the presence of Fib [43]. It is important to note that the concentrations used were established based on the content of PAni reported by previous research. For example, the PAni cytotoxicity results for fibroblast NIH/3T3 cells [44] and macrophage cells [45] are similar to previous works.

The PAni homopolymer presents content-dependent cytotoxicity to NIH/3T3 cells, noting a moderate to severe effect at the contents of 1 and 2 mg/mL, respectively. On the contrary, severe effects were noted at the PAni contents of 0.03 and 0.2 mg/mL. The differences in PAni toxicity could be associated with the composition and morphology of PAni. Fib-NH_2_ presents a severe cytotoxicity event at 0.33 mg/mL, due to the substituted aniline, which could react with the cells.

Fib-*g*-PAni 1:1 and Fib-*g*-PAni 1:0.5 showed a similar pattern with content-dependent cytotoxicity, except at the lowest content (0.1 mg/mL), where moderate toxicity was noted. This result could be explained by the excellent dispersion of PAni under this condition, favoring a closer interaction with cells. From 0.5 to 2 mg/mL of the Fib-*g*-PAni copolymers, no toxicity or mild toxicity was noted, and at 4 mg/mL, moderate toxicity was observed. The low cytotoxic effect of the copolymers contrasted with the highly marked cytotoxic effect of Fib-NH_2_ and PAni.

Generally, the Fib-*g*-PAni from 0.5 to 2 mg/mL could be considered biocompatible with cells. The Fib-*g*-PAni 1:1 presents higher cellular viability; these results are attributed to the fact that at lower contents, the interaction and probable internalization in cells is favored, which could be toxic for the cells; however, at higher contents, the molecules agglomerate, reducing the interaction with cells [46]. These results correspond to a comparable copolymer of Fib/PAni core/shell coaxial fiber-type, which exhibited good biocompatibility, cell attachment, and proliferation of L929 fibroblast cells [47]. Consequently, based on the results, the copolymers could be a promising material for biomedical applications.

## 4. Conclusions

Silk fibroin modification was performed through one-pot synthesis with 3-aminobenzoic acid esterification and subsequently with polyaniline grafting by oxidative polymerization. The one-pot methodology for grafting PAni chains onto fibroin structure provides a synthetic method that is easy to scale. The morphological study showed that each modification process changes the material structure, which was also reflected in the chemical structure and thermal stability. The silk fibroin:polyaniline mass ratio also showed a significant effect, mainly in optical properties and electroactivity. These differences are related to a higher degree of polymerization resulting from a higher polyaniline content, accentuating both properties. As a first approach, the cytotoxicity results for copolymers are promising; nevertheless, more experiments will be carried out, e.g., increasing the exposure time of fibroblasts to the copolymers, and increasing the concentration, among others. Based on the results of this research and the already known properties of fibroin, it can be expected that the graft copolymer is suitable as a biocompatible platform for developing electrochemical sensors that can be in contact with living tissue.

## Figures and Tables

**Table 1:** Formulations employed for fibroin-*g*-PAni (Fib-*g*-PAni).

Sample	Fib (g)	3-Aminobenzoic Acid/g (mmol)	Anilinium Sulfate/g (mmol)	APS/g (mmol)	Yield/%
Fib-*g*-Pani 1:0.1	1	0.15 (1.1)	0.10 (0.523)	0.149 (0.654)	15%
Fib-*g*-Pani 1:0.25	1	0.15 (1.1)	0.25 (1.307)	0.373 (1.634)	45%
Fib-*g*-Pani 1:0.5	1	0.15 (1.1)	0.50 (2.615)	0.746 (3.269)	87%
Fib-*g*-Pani 1:1	1	0.15 (1.1)	1.00 (5.230)	1.492 (6.537)	95%

## Data Availability

Not applicable.
